# Supplementary material for: Development and External Validation of Deep-Learning-Based Tumor Grading Models in Soft-Tissue Sarcoma Patients Using MR Imaging
Source: Cancers (Basel). 2021 Jun 8;13(12):2866. doi: 10.3390/cancers13122866 (PMC8227009; doi:10.3390/cancers13122866)
Supplement: Supplementary file 1 [file cancers-13-02866-s001.zip › cancers-1217589-supplementary.pdf]

Supplemental Material

Supplemental Figures 2
Figure S1 Patient Workflow 2
Figure S2 Calibration Curves: 3
Supplemental Tables 4
Table S1 Histologies of Soft-Tissue Sarcomas 4
Table S2 STARD Checkliste 5
Table S3 MRI acquisition parameters 7

Supplemental Figures

Figure S1 Patient Workflow

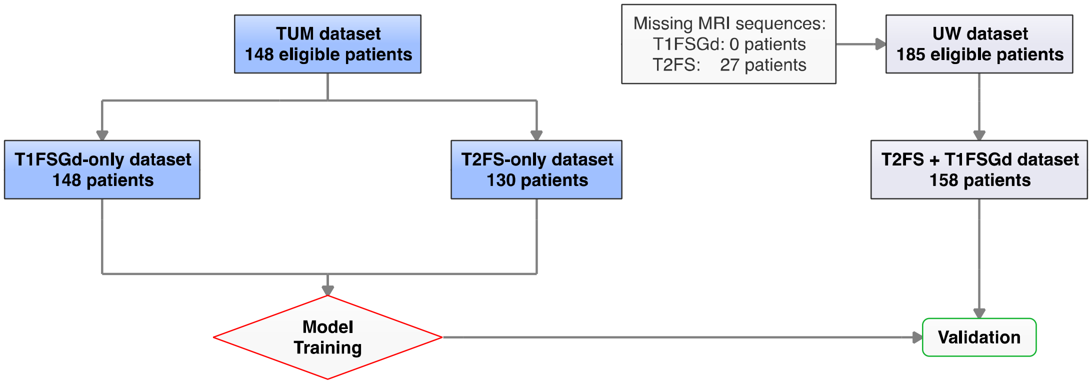

Figure S2 Calibration Curves

Calibration curves for the *Clinical* model (a), the *Tumor-Volume* model (b), *Clinical-Volume-combined* (c), the *T1FSGd* model (d), and the *T2FS* (e) on the test set are shown.

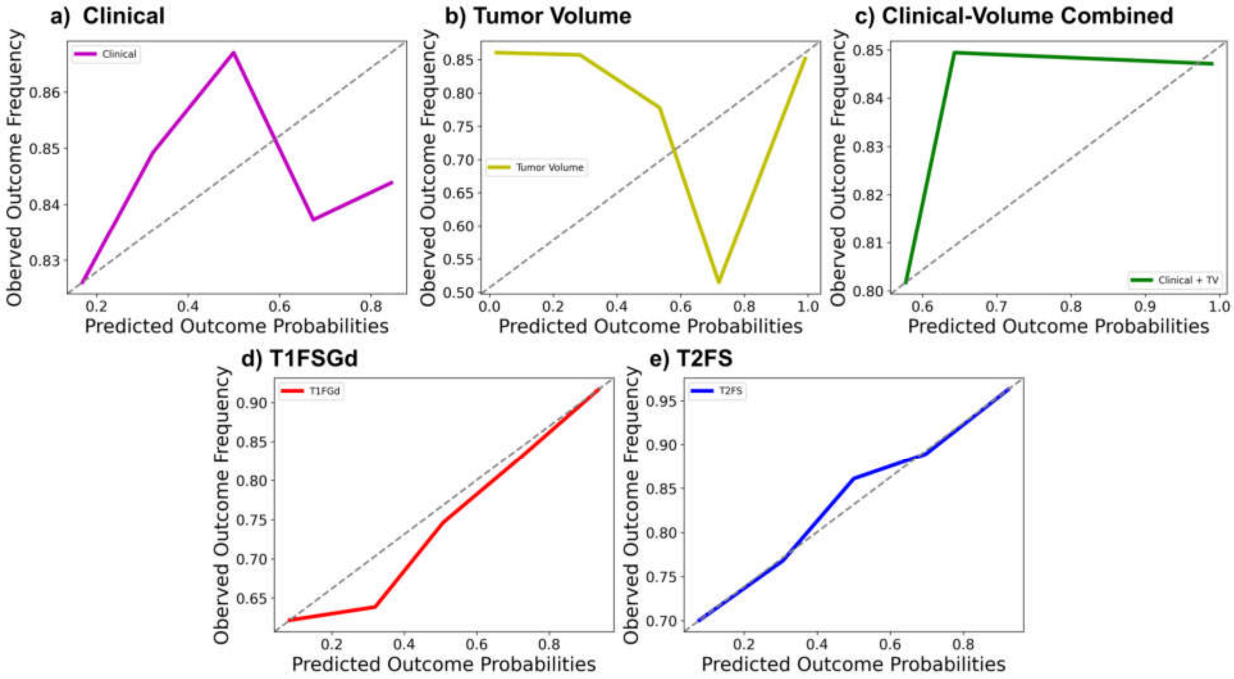

Supplemental Tables

Table S1 Histologies of Soft-Tissue Sarcomas

The distribution of histologies was significantly different (p-value <0.001, Fisher's exact test). Sorted by patient number in the TUM cohort.

Histological subtype (%)	TUM (Test cohort)	UW (Training cohort)
**Liposarcoma[1]**	42 p (28 %)	4 p (2 %)
**Pleomorphic sarcoma**	31 p (21 %)	56 p (35 %)
**Myxofibrosarcoma**	19 p (13 %)	9 p (6 %)
**Synovial sarcoma**	13 p (9 %)	10 p (6 %)
**Myxoid liposarcoma**	11 p (7 %)	18 p (11 %)
**Dedifferentiated liposarcoma**	10 p (7 %)	3 p (2 %)
**Leiomyosarcoma**	7 p (5 %)	15 p (9 %)
**Spindle cell sarcoma**	3 p (2 %)	11 p (7 %)
**Rhabdomyosarcoma**	2 p (1 %)	3 p (2 %)
**Pleomorphic liposarcoma**	2 p (1 %)	3 p (2 %)
**Angiosarcoma**	2 p (1 %)	1 p (1 %)
**Alveolar soft part sarcoma**	2 p (1 %)	0 p (0 %)
**Myofibrosarcoma**	1 p (1 %)	4 p (3 %)
**Fibromyxoid sarcoma**	1 p (0 %)	3 p (2 %)
**Fibrosarcoma**	1 p (1 %)	1 p (1 %)
**No classification**	1 p (1 %)	1 p (1 %)
**Malignant peripheral nerve sheath tumor**	0 p (0 %)	6 p (4 %)
**Myxoinflammatory fibroblastic sarcoma**	0 p (0%)	3 p (2%)
**Extraskeletal myxoid Chondrosarcoma**	0 p (0 %)	3 p (2 %)
**Solitary fibrous tumor**	0 p (0 %)	2 p (1 %)
**Epitheloid angiosarcoma**	0 p (0 %)	1 p (1 %)
**Small round cell sarcoma**	0 p (0 %)	1 p (1 %)

[1] not further specified.

Table S2 STARD Checkliste

Checklist following the Standards for Reporting of Diagnostic Accuracy Studies (STARD) recommendations. [1]

Section & Topic	No	Item	Reported on page #
**TITLE OR ABSTRACT**			
	**1**	Identification as a study of diagnostic accuracy using at least one measure of accuracy<br>(such as sensitivity, specificity, predictive values, or AUC)	2
**ABSTRACT**			
	**2**	Structured summary of study design, methods, results, and conclusions<br>(for specific guidance, see STARD for Abstracts)	2
**INTRODUCTION**			
	**3**	Scientific and clinical background, including the intended use and clinical role of the index test	3
	**4**	Study objectives and hypotheses	3
**METHODS**			
Study design	**5**	Whether data collection was planned before the index test and reference standard<br>were performed (prospective study) or after (**retrospective study**)	3
Participants	**6**	Eligibility criteria	3,4
	**7**	On what basis potentially eligible participants were identified (such as symptoms, results from previous tests, inclusion in registry)	3,4
	**8**	Where and when potentially eligible participants were identified (setting, location and dates)	3,4, Table1
	**9**	Whether participants formed a **consecutive**, random or convenience series	3
Test methods	**10a**	Index test, in sufficient detail to allow replication	4-6
	**10b**	Reference standard, in sufficient detail to allow replication	7
	**11**	Rationale for choosing the reference standard (if alternatives exist)	
	**12a**	Definition of and rationale for test positivity cut-offs or result categories<br>of the index test, distinguishing pre-specified from exploratory	6
	**12b**	Definition of and rationale for test positivity cut-offs or result categories<br>of the reference standard, distinguishing pre-specified from exploratory	6
	**13a**	Whether clinical information and reference standard results were available<br>to the performers/readers of the index test	Not applicable
	**13b**	Whether clinical information and index test results were available	Not applicable

		to the assessors of the reference standard	
Analysis	**14**	Methods for estimating or comparing measures of diagnostic accuracy	6
	**15**	How indeterminate index test or reference standard results were handled	Not applicable
	**16**	How missing data on the index test and reference standard were handled	Not applicable
	**17**	Any analyses of variability in diagnostic accuracy, distinguishing pre-specified from exploratory	6
	**18**	Intended sample size and how it was determined	Not pre-specified
**RESULTS**			
Participants	**19**	Flow of participants, using a diagram	Fig S1
	**20**	Baseline demographic and clinical characteristics of participants	Table 1
	**21a**	Distribution of severity of disease in those with the target condition	Table 1
	**21b**	Distribution of alternative diagnoses in those without the target condition	Not applicable
	**22**	Time interval and any clinical interventions between index test and reference standard	Not applicable
Test results	**23**	Cross tabulation of the index test results (or their distribution) by the results of the reference standard	7
	**24**	Estimates of diagnostic accuracy and their precision (such as 95% confidence intervals)	7,8
	**25**	Any adverse events from performing the index test or the reference standard	Not applicable
**DISCUSSION**			
	**26**	Study limitations, including sources of potential bias, statistical uncertainty, and generalisability	13
	**27**	Implications for practice, including the intended use and clinical role of the index test	13
**OTHER INFORMATION**			
	**28**	Registration number and name of registry	Not applicable
	**29**	Where the full study protocol can be accessed	Not applicable
	**30**	Sources of funding and other support; role of funders	13

Table S3 MRI acquisition parameters

Median values are depicted. The range is noted in brackets.

	Sequence	TUM[a] Training	UW[b] Validation
**Magnetic field strength**		1.5 T (1.0 - 3.0)	1.5 (0·7 - 3·5)
**In-Plane resolution (mm)**	**T1FSGd**	0·78 x 0.78 (0·27 x 0·27 -1·4 x 1·4)	0.56 x 0.56 (0.19 x 0.19 - 1.56 x 1.56)
**Slice thickness (mm)**		5.82 (3.29 - 10)	6 (1.0 - 12.0)
**Matrix**		320 x 320 (176 x 176 - 5121 x 1152)	512 x 512 (204 x 176 - 1200 x 1200)
**TR (ms)**		644 (6 - 1311)	623 (3.98 - 1533)
**TE (ms)**		12 (4 - 1450)	12 (0.3 - 40)
**In-Plane resolution (mm)**	**T2FS**	0.9 x 0.9 (0.31x0.31 - 1·75x1·75)	0.625 x 0·625 (0.17 x 0.17 - 1.71 x 1.71)
**Slice thickness (mm)**		7 (3.3 - 15)	6 (3.0 - 11.9)
**Matrix**		448 x448 (176 x 224 - 1536 x 1536)	480 x 480 (192 x 160 - 880 x880)
**TR (ms)**		6830 (1086 - 18516)	3900 (4.95 -13000)
**TE (ms)**		60 (16 - 133)	68.4 (2.49-738)

Abbreviation: p: patients, T1wfs: T1-weight fat saturated, T2wfs: T2-weight fat saturated, TE: echo time, TR: repetition time.

[a]MRI Scanner: GE (Chicago, USA): Signa; Philips (Amsterdam, Netherlands): Achieva, Ingenia; Siemens (Munich, Germany): Verio, Avanto, Symphony.

[b]MRI Scanner: GE (Chicago, USA): Discovery MR750, Signa, Optima 450; Hitachi (Tokyo, Japan): Oasis; Philips (Amsterdam, Netherlands): Achieva, Gyroscan NT, Ingenia, Intera; Siemens (Munich, Germany): Avanto, Area, Espree, Harmony, Symphony, TrioTim; Toshiba (Tokyo, Japan): Titan.

References

[1] Bossuyt PM, Reitsma JB, Bruns DE, Bruns DE, Glasziou PP, Irwig L, et al. STARD 2015: An updated list of essential items for reporting diagnostic accuracy studies1. Radiology 2015;277:826–32. doi:10.1148/radiol.2015151516.
